# The Prevalence and Correlates of Depression, Anxiety, and Insomnia among Camp Residing Palestinian Women Migrants during the Outbreak of the War on Gaza: A Cross-Sectional Study from Jordan

**DOI:** 10.3390/medicina60081228

**Published:** 2024-07-29

**Authors:** Omar Gammoh, Bilal Sayaheen, Mervat Alsous, Ahmed Al-Smadi, Bilal Al-Jaidi, Alaa A. A. Aljabali

**Affiliations:** 1 Department of Clinical Pharmacy and Pharmacy Practice, Faculty of Pharmacy, Yarmouk University, Irbid 21163, Jordan; mervat.alsous@yu.edu.jo; 2 Department of Translation, Yarmouk University, Irbid 21163, Jordan; bsayaheen@yu.edu.jo; 3 Faculty of Nursing, Al al-Bayt University, Mafraq 25113, Jordan; 4 Department of Medicinal Chemistry and Pharmacognosy, Faculty of Pharmacy, Yarmouk University, Irbid 21163, Jordan; bilal.aljaidi@yu.edu.jo; 5 Department of Pharmaceutics and Pharmaceutical Technology, Faculty of Pharmacy, Yarmouk University, Irbid 21163, Jordan; alaaj@yu.edu.jo

**Keywords:** Gaza war, women, Palestinian, refugees, mental health, Jordan

## Abstract

*Background and Objectives*: The current war on the Gaza strip and the circulating violent content is believed to negatively impact the mental health of the Palestinians living in refugee camps outside their homeland. This study explores the prevalence and correlates of depression, anxiety, and insomnia in a cohort of female Palestinian refugees in Jordan who have family members entangled in the persistent conflict in the Gaza strip. *Materials and Methods*: This cross-sectional study employed validated tools to assess depression, anxiety, and insomnia in women residing in a Gaza camp located in Jerash, Jordan. The correlates were determined by regression analysis. *Results*: The study unearths disconcerting statistics from 177 recruited women, revealing alarmingly high rates of severe depression (73%), anxiety (60%), and insomnia (65%). Multivariable analysis revealed that severe depression was significantly associated with prior diagnosis with chronic diseases (OR = 3.0, CI = 1.36–6.58), and having a first-degree relative in Gaza (OR = 0.42, CI = 0.20–0.85). Additionally, severe insomnia was associated with “losing relatives or friends in the war” (OR = 3.01, CI = 1.41–6.44), and “losing connection with families and friends” (OR = 3.89, CI = 1.58–9.53). *Conclusions*: The implications of these results are profound, underscoring the immediate and imperative need for both medical and psychiatric interventions aimed at addressing the substantial psychological burden borne by this population because of the ongoing conflict.

## 1. Introduction

Palestine was under the control of the Ottoman Empire from 1517 until the First World War [1]. Since 1900, waves of Jewish immigration to Palestine notably increased due to the hard situation suffered by Jewish people in Europe and their desire to establish a Jewish state in Palestine. This immigration was met with a certain level of discomfort and unease, as they were not seen as an integral part of the Zionist movement. According to [2] (p. 1) ‘it has in fact been less than a century since Jews and Arabs began to view one another as enemies’. An important historical turning point that fueled anger amongst Arabs and Palestinians and immensely increased tension was the letter sent by Lord Arthur Balfour on 2 November 1917 to the leader of British Jewry, Lord Rothschild, which approved the establishment of a national home for the Jewish people in Palestine.

The ‘Balfour Declaration’, in addition to the British mandate, served as the cornerstone in shaping the beginning of the Arab–Israeli dispute and the Palestinian–Israeli conflict, leading to the founding of a state or homeland for Jews in Palestine i.e., the state of Israel in Palestine [3]. The Arabs considered the Balfour Declaration to constitute a threat to their identity and future, one that violated their rights to their land and threatened their existence and property in their homeland, Palestine [4,5,6].

Since then, the relationship between Arabs and Israel has gone through major upheavals, moving back and forth between waging wars and pursuing peace. In 1947 The United Nations proposed the termination of the British mandate in Palestine and at the same time partitioning Palestine into two states: one for the Jews and the other for the Palestinians. Nevertheless, the resolution was not implemented due to Palestinian rejection and the escalation of the conflict. Immediately after this proposed resolution in 1948, Israel occupied the majority of the Palestinian land, a catastrophe known in the Arab world as Nakba. The Nakba had fatal consequences for Palestinians, as hundreds of thousands were forced to leave their homeland for neighboring countries, mostly to Jordan. As a result, Palestinians became dispersed across many countries and regions around the world, where diaspora and asylum constitute a large part of the Palestinian experience. Moreover, in 1967 another war erupted between Israel and several Arab countries (Jordan, Egypt, Iraq, and Syria). The outcomes of the 1967 war were also catastrophic for the Arab world as the war ended with Israel occupying and taking control of new territories, including the Egyptian Sinai Peninsula, the Gaza Strip, and the West Bank, which were under Jordanian control, in addition to the Golan Heights, which were under Syrian control. The suffering and oppression of the Palestinians have increased immensely after the Arab defeat in the 1967 war [7]. Palestinian suffering due to the Israeli occupation of their homeland is represented in various forms, including Human rights violations against Palestinians, arbitrary arrests, the dispossession of Palestinian lands, and the economic blockade of the Gaza Strip. Palestinian resistance to the Israeli occupation took various forms, starting with popular protests and clashes, and turning into a military conflict known as the Intifada (both the first Intifada in 1987–1993 and the second Intifada in 2000–2005) [8].

In 2005, Israeli occupation forces withdrew from the Gaza Strip and evacuated the settlements that were built there. Since then, Israeli occupation forces have carried out several military operations in the strip. Indeed, some of these operations turned into wars that lasted weeks and left thousands of martyrs. The Islamic Resistance Movement (Hamas) took control of the Gaza Strip in June 2007, Israel then, in September 2007, declared Gaza a “hostile entity” and, in October of the same year, imposed a comprehensive siege on it.

On 7 October 2023 Hamas and Palestinian Islamic Jihad, and as a form of resistance against the occupation and its continuous violations against Palestinians, launched a major attack from the Gaza Strip. The response came in the form of the bombardment of entire neighborhoods, hospitals, schools, and public institutions, resulting in thousands of martyrs and injuries, most of them children and women, and destroying hundreds of homes, schools, mosques, churches, hospitals, and public institutions.

Although the current war on Gaza has had a huge psychological impact worldwide, this impact is hypothesized to be more detrimental among the relatives of the people of Gaza and particularly among women, who are more vulnerable to psychological distress, namely depression, anxiety, and insomnia.

Based on the definition of the Diagnostic and Statistical Manual of Mental Disorders, Fifth Edition (DSM-5), the most accurate diagnosis of major depression requires the presence of at least five symptoms within a preceding period of two weeks. The major symptoms present should encompass, at least, a depressed mood or a loss of interest. Minor or secondary symptoms include changes in weight, appetite, sleeping difficulties (insomnia or hypersomnia), fatigue, lack of concentration, feelings of guilt, and suicide [9]. Anxiety, according to the DSM-5 is a condition characterized by excessive, uncontrollable worry (apprehension), accompanied by restlessness, irritability, tension, and sleep disturbance [10]. Insomnia is characterized by difficulty finding sleep, maintaining quality sleep, and early waking. Together, the above-mentioned symptoms are more-often present in refugees [11].

The crosstalk between mental health and chronic diseases is well established, especially in war-displaced refugees. The long-term post-migration stress accompanied by inflammation provides a common ground for long-term physical diseases such as diabetes and hypertension and of mental disorders such as depression and anxiety. For example, one in every two refugees escaping from wars reporting depressive and traumatic symptoms were also diagnosed with cardiovascular diseases [12]. In addition, their unhealthy lifestyle could be related to the exacerbation of both chronic diseases and mental health. For example, one study underscored the way in which the lack of physical exercise was significantly associated with severe depression, anxiety, and insomnia in refugees residing in Jordan [13].

According to estimations, 18% of Palestinian refugees reside in camps that have poor living conditions when compared with non-camp settings. The high population density and the inadequate educational, social, and medical infrastructure mean that the residents of these camps become extremely fragile [14].

The research concerning mental health epidemiology in Palestinians is limited; for example, one study conducted before the war concluded that 52% of the people living in the Gaza Strip reported different sleeping problems [15], while another study in the West Bank demonstrated that 50% of Palestinians experience severe depression and 19% experienced severe anxiety. Taken together, these reported symptoms significantly affected their quality of life [16,17]. After the start of the October 7th war, limited data have been made available regarding the mental health status of Palestinians, especially those living outside Palestine who have relatives in Gaza. The current research asks the following: what is the prevalence of depression, anxiety, and insomnia among Palestinian women migrants residing in the Gaza camp of Jerash in the light of the ongoing war in their homeland, and what are the correlates of these symptoms?

Therefore, the present study sought to examine the prevalence and correlates of depression, anxiety, and insomnia in a cohort of female Palestinian refugees in Jordan who have relatives in the Gaza Strip during the outbreak of the war on Gaza. The authors hypothesize that all of the residents of the Gaza camp report a high burden of depression, anxiety, and insomnia. Additionally, the authors hypothesize that losing close relatives in this war could be associated with a severe mental health burden.

## 2. Materials and Methods

### 2.1. Study Design

This is a cross-sectional cohort study using pre-defended inclusion criteria sampling for Palestinian female refugees residing in the Gaza camp in Jerash, Jordan.

### 2.2. Recruitment

In this study, female refugees attending social and health clinics in the Gaza camp for refugees were invited to participate. The study tool was uploaded to a Google form and a generated link was sent to the interested and eligible participants on their smartphones.

The ethical rights of all participants to withdraw from the study at any time were strictly upheld. The data collection for this research was conducted from 10–29 January 2024, as part of a larger project aimed at evaluating the mental health of refugees residing in Jordan. The research was granted ethical approval from University Institutional Review Board (IRB) number 692/2023. The sizing of our sample cohort drew inspiration from insights gleaned from a prior investigation spearheaded by Alduraidi and Waters in 2018. The choice of this reference is underpinned by its direct alignment with our research objectives and the striking parallels shared between their study and ours using a sample size of 177 participants [18].

### 2.3. Inclusion Criteria

Our study’s inclusion parameters incorporate Palestinian women refugees who are currently situated in the Gaza refugee camp in Jordan, and who still have family and or friends in their homeland, the Gaza Strip.

### 2.4. Exclusion Criteria

Women who are not Palestinian refugees, or those not residing in the Gaza camp of Jerash were excluded from the study.

### 2.5. Study Instrument

#### Covariates

We methodically crafted a self-administered, thoroughly structured online survey to collect perceptions from our participants. This tool was considerately intended to include a wide collection of demographic information, which incorporated age, marital status, educational accomplishment, employment, smoking behaviors, the presence of chronic illnesses (such as hypertension and type 2 diabetes), and the utilization of chronic medications. Extending beyond this, the survey was thoughtfully designed to specifically investigate our participants’ influence on Gaza. In this activity, ‘yes’ or ‘no’ questions are used to query the existence of first-degree relatives, second-degree relatives, or close friends living in Gaza. Furthermore, we inquired into personal encounters linked to the current conflict, incorporating an exploration of family members or friends who may have been directly impacted or lost their lives amid these attacks. Furthermore, our survey ventured into the domain of sleep-related distributions, investigating the contributors’ occurrences over the preceding two weeks.

### 2.6. Outcome Measures

#### 2.6.1. Depression

In our pursuit to indicate the intensity of depressing symptoms among our participants, we opted for the widely recognized Patient Health Questionnaire-9 (PHQ-9). It is worth noting that the Arabic-authenticated iteration of this survey holds accurately to the criteria allocated in the Diagnostic and Statistical Manual of Mental Disorders-IV [19]. The PHQ-9, an accurately designed tool, assesses symptoms of depression over the previous two weeks and exposes a grading scale that spans from 0 to 27. Remarkably, a score surpassing 14 on this scale serves as a reliable indication of severe depression [20,21,22]. This points out its significance as a robust tool (Cronbach alpha = 0.88) for evaluating the severity of depressive symptoms within the context of our study.

#### 2.6.2. Anxiety

In our venture to determine the extent of anxiety among our participants, we employed the General Anxiety Disorder-7 (GAD-7). This self-administered scale is adept at evaluating numerous anxiety symptoms faced over the preceding two months, encompassing a concise set of seven questions. With a maximum score of twenty-one, a threshold exceeding 14 implies the presence of severe anxiety (Cronbach alpha = 0.95) [13,21,23].

#### 2.6.3. Insomnia

To assess the severity of insomnia within our study cohort, we employed the Insomnia Severity Index Arabic version (ISI-A). This scale, initially devised by Morin et al. (Morin, 1993), [24], comprises seven questions and yields a maximum score of 28. A score greater than 14 serves as a noteworthy indicator of severe insomnia symptoms. Importantly, the ISI-A has undergone validation for use in the Arabic language (Cronbach alpha = 0.84), featuring its reliability and relevance in our research [25].

### 2.7. Data Analysis

Frequencies and percentages were used to describe the participants’ demographics and the prevalence of depression, anxiety, and insomnia in the study sample. To identify which factors are independently associated with the outcome variables, a preliminary univariate logistic regression analysis was carried out to identify potentially significant covariates (*p* < 0.1) followed by a multivariate logistic regression analysis for each one of the outcome variables (depression, anxiety, and insomnia). Statistical significance was set at 2-sided *p* < 0.05 and estimates were set at 95% CI. Data were analyzed using SPSS software (Version 21).

## 3. Results

### 3.1. Sample Characteristics

Data were analyzed from 177 females, 94 participants (53.1%) were aged above 35 years old, 107 (60.5%) were married, 97 (54.8%) received primary and secondary education, 135 (76.3%) were unemployed, 152 (87.4%) were non-smokers, 93 (52.5%) had first-degree relatives in Gaza, 58 (32.8%) had a second-degree relative in Gaza, 88 (49.7%) had lost a relative or a friend in Gaza in the preceding week, and 47 (26.6%) had lost connection with a relative or a friend in the preceding week, as shown in Table 1.

### 3.2. The Prevalence of Depression, Anxiety, and Insomnia

According to the PHQ-9 scale, 129 participants (73%) reported a score above the threshold for severe depression. In addition, according to the GAD-7 scale, a total of 105 participants (59.3%) reported a score corresponding to severe anxiety, and, based on the ISI-A scale, 114 participants (64.6%) reported a score corresponding to severe insomnia, as shown in Table 2.

### 3.3. Correlates of Depression, Anxiety, and Insomnia

The multivariate logistic regression for depression as the dependent variable was finally adjusted for “diagnosis with chronic diseases” and “having a first-degree relative in Gaza”. The model revealed that severe depression was significantly associated (OR = 3.00, 95% CI = 1.36–6.58, *p* = 0.006) with “diagnosis with chronic diseases” and “having a first-degree relative in Gaza” (OR = 0.42, 95% CI = 0.20–0.85, *p* = 0.01). The multivariate logistic regression for anxiety as the dependent variable revealed no associations with any of the covariates investigated.

The multivariate logistic regression for insomnia as the dependent variable was finally adjusted for “I lost one of my relatives or friends in the war” and “I lost connection with my relatives or friends in the war”. The model revealed that severe insomnia symptoms were significantly associated (OR = 3.01, 95% CI = 1.41–6.44, *p* = 0.004) with “losing one of the relatives or friends” and “losing connection with relatives and friends” (OR = 3.89, 95% CI = 1.58–9.53, *p* = 0.003), as shown in Table 3.

## 4. Discussion

The present study aimed to investigate the prevalence and determinants of depression, anxiety, and insomnia among Palestinian women refugees in Jordan who have relatives enduring conflict in Gaza. Our findings reveal an unprecedentedly high prevalence of severe depression, affecting up to 73% of the study sample. This high burden is significantly associated with the diagnosis of chronic diseases and having first-degree relatives in Gaza. While previous research has estimated the rate of severe depression in Palestinian refugees in Jordan at 42% [18], the extraordinary rate in our study can be explained by several factors. First, depression was assessed over the preceding two weeks, coinciding with the war in Gaza. In our study, all selected participants were female, and each had ties to Gaza, making them eyewitnesses to the dreadful experiences of war, incorporating scenes of mutilated bodies, bombarded homes, and injured children. Such encounters place an immense psychological burden on individuals. Additionally, current evidence highlights the heightened vulnerability of females to depression [26,27].

Our research uncovered a significant connection between chronic diseases and the severity of depression, which aligns with prior studies on refugees where health conditions were intricately connected with depressive symptoms [28,29]. This correlation can be processed by the shared pathological factors of inflammation and hormonal imbalances that trigger chronic diseases, such as hypertension and type 2 diabetes [30,31,32]. This result underlines the importance of studying the physical health of individuals in the context of mental wellbeing.

Furthermore, our results emphasize the prevalence of severe anxiety symptoms in approximately 60% of the sample. It is noteworthy that females exhibit a greater susceptibility to anxiety compared with men, as confirmed by previous extensive research, with females being nearly twice as likely to develop anxiety disorders [33]. Moreover, it is well-documented that anxiety disorders are more regularly reported among refugees when compared with non-displaced populations [34,35]. Additionally, our findings regarding anxiety indicators assessed over the past two weeks coincide with the onset of conflicts affecting the relatives of our study participants. This temporal alignment sheds light on the immediate impact of the conflict on the mental wellbeing of these individuals. The link between such complex factors in the context of a conflict-affected population highlights the difficulties of mental health and the importance of considering multiple perspectives.

We found that severe insomnia affected 65% of the study sample. Notably, this was significantly associated with two key factors: the loss of a relative or friend in the war and the loss of contact with relatives and friends. The emotional and psychological wellbeing of individuals can be profoundly impacted when they lose family members or close friends [36]. Previous studies have shown that exposure to traumatic incidents related to war increases the risk of developing post-traumatic stress disorder [37]. Insomnia, characterized by difficulties in initiating or maintaining sleep, has been linked to the loss of friends or relatives [38]. Insomnia can have a detrimental effect on an individual’s daily activities, mood, and cognitive function [39]. This research effort stands as a timely and emotional response to the profound violence imposed upon civilians in the Gaza Strip throughout times of war. While gathering explicit data from the civilians of Gaza remains a remarkable challenge, it is of vital significance to demonstrate the psychological burden conveyed by their relatives. The study draws on its strength from several commendable facets, including the careful assembly of a representative sample, the thoughtful utilization of justified scales for assessment, and the prompt completion of the study. These elements contribute to the strength and timeliness of our findings.

Therefore, we believe that the findings of our study pave the way for additional studies that can provide health care providers, health policymakers, and stakeholders with insights to enhance mental health awareness for this community that has been based in Jerash since 1968, to address their mental and social wellbeing requirements and to address the potential risk factors in an attempt to alleviate their psychiatric burden and to improve their quality of life. For example, the integration of mental health clinics in primary care centers could serve patients with chronic diseases who are more vulnerable to mental health distress. Additionally, awareness campaigns in the camp to overcome stigma towards mental health, and expanding the human capacity by educating health care providers such as nurses and pharmacists to screen and guide patients to proper psychiatric care are suggested strategies.

However, it is equally important to acknowledge the limitations inherent in our study. These limitations may incorporate factors such as the nature of self-reported data, the potential for selection bias within the sample, and the limitations associated with the use of standardized scales in capturing the full range of participants’ experiences. These limitations serve as proof of the balanced and thorough approach fundamental to this research. The cross-sectional design, while revealing, does not allow for the monitoring of psychiatric indications over time intervals. Additionally, the lack of specialized psychiatrists for identifying psychiatric illnesses may impact the precision of the diagnoses. In addition, the assessment of post-traumatic stress disorder (PTSD) symptoms was not assessed in the current study. PTSD represents a major mental health complaint for this fragile population exposed to the continuous flow of circulating violent war content. Our future studies will focus on estimating PTSD and identifying its correlates. Looking ahead, our future steps should involve gaining access to Gaza to directly assess the profound psychiatric outcomes among the civilians who endured this war. This will provide a more comprehensive understanding of the long-term effects and inform future interventions.

## 5. Conclusions

In conclusion, our study highlights the substantial psychiatric burden borne by female Palestinian refugees residing in Jordan, particularly within the context of the ongoing conflict in the Gaza Strip. The prevalence of severe depression, anxiety, and insomnia among this population demands immediate and focused attention. Our findings underscore the imperative for expeditious medical and psychiatric interventions to mitigate the profound impact of the current conflict. Prioritizing the mental health and wellbeing of these refugees is of utmost importance.

In summary, our research underscores the urgency of this matter and underscores the critical need for swift, targeted interventions to support these women in their path toward recovery.

## Figures and Tables

**Table 1:** Study sample characteristics (*n* = 177).

Factor	Category	*n* (%)
Age	Below 35 years	83 (46.9)
	35 years and above	94 (53.1)
Marital status	Single	70 (39.5)
	Married	107 (60.5)
Education level	Primary and high school	97 (54.8)
	University education	80 (45.2)
Employment status	Unemployed	135 (76.3)
	Employed	42 (23.7)
Smoking status	Non-smokers	152 (87.4)
	Smokers	22 (12.6)
Diagnosed with chronic diseases		66 (37.3)
Using chronic medications		73 (41.2)
I have a first-degree relative in Gaza		93 (52.5)
I have a second-degree relative in Gaza		58 (32.8)
I have a close friend in Gaza		24 (13.6)
I lost one of my relatives or friends in the war		88 (49.7)
I lost connection with my relatives or friends in the war		47 (26.6)

**Table 2:** The prevalence of depression, anxiety, and insomnia.

Outcome Variable	Category	*n* (%)
Severe depression (PHQ-9)	Below threshold	48 (27.1)
	Above threshold	129 (72.9)
Severe anxiety (GAD-7)	Below threshold	72 (60.7)
	Above threshold	105 (59.3)
Severe insomnia (ISI-A)	Below threshold	63 (35.6)
	Above threshold	114 (64.6)

Depression was assessed using the Patient Health Questionnaire-9 (PHQ-9) using a cut-off value of above 14 for severe depression. Anxiety was measured using the General Anxiety Disorder-7 (GAD-7) using a cut-off value of above 14 for severe anxiety. Insomnia was assessed using the Insomnia-Severity Index-Arabic version (ISI-A) using a cut-off value of above 14 for severe insomnia.

**Table 3:** Univariate and multivariate logistic regression analysis for the three outcome variables.

**Severe Depression (PHQ-9)**						
	**Univariate Analysis**			**Multivariate Analysis**		
	**OR**	**95% CI**	***p***	**OR**	**95% CI**	***p***
Covariates						
Age	1.31	0.69–2.58	0.39			
Marital status	1.00	0.51–1.97	0.99			
Education level	0.97	0.49–1.88	0.92			
Employment status	0.91	0.42–1.96	0.81			
Smoking status	0.92	0.33–2.51	0.33			
Diagnosed with chronic diseases	2.50	1.17–5.33	0.01	3.00	1.36–6.58	0.006 *
Using chronic medications	1.39	0.70–2.78	0.34			
I have a first-degree relative in Gaza	0.51	0.26–1.07	0.05	0.42	0.20–0.85	0.01 *
I have a second-degree relative in Gaza	1.93	0.90–4.13	0.09			
I have a close friend in Gaza	1.13	0.42–3.05	0.81			
I lost one of my relatives or friends in the war	1.75	0.89–3.44	0.10			
I lost connection with my relatives or friends in the war	1.30	0.60–2.82	0.51			
**Severe Anxiety (GAD-7)**						
	**Univariate Analysis**			**Multivariate Analysis**		
	**OR**	**95% CI**	***p***	**OR**	**95% CI**	***p***
Covariates						
Age	1.23	0.67–2.25	0.49			
Marital status	1.16	0.63–2.14	0.63			
Education level	0.79	0.43–1.45	0.45			
Employment status	0.61	0.31–1.22	0.16			
Smoking status	1.51	0.58–3.93				
Diagnosed with chronic diseases	1.20	0.64–2.25	0.56			
Using chronic medications	1.17	0.64–2.17	0.60			
I have a first-degree relative in Gaza	0.81	0.48–1.49	0.51			
I have a second-degree relative in Gaza	1.65	0.86–3.18	0.13			
I have a close friend in Gaza	0.79	0.33–1.86	0.58			
I lost one of my relatives or friends in the war	1.30	0.71–2.37	0.39			
I lost connection with my relatives or friends in the war	1.29	0.65–2.57	0.46			
**Severe Insomnia (ISI-A)**						
	**Univariate Analysis**			**Multivariate Analysis**		
	**OR**	**95% CI**	***p***	**OR**	**95% CI**	***p***
Covariates						
Age	1.28	0.68–2.36	0.44			
Marital status	1.24	0.66–2.32	0.51			
Education level	0.86	0.46–1.59	0.63			
Employment status	0.89	0.42–1.77	0.69			
Smoking status	1.98	0.69–5.66	0.20			
Diagnosed with chronic diseases	1.17	0.61–2.23	0.63			
Using chronic medications	0.82	0.44–1.52	0.52			
I have a first-degree relative in Gaza	0.67	0.36–1.26	0.22			
I have a second-degree relative in Gaza	1.35	0.69–2.63	0.38			
I have a close friend in Gaza	1.12	0.45–2.79	0.80			
I lost one of my relatives or friends in the war	1.53	0.83–2.85	0.10	3.01	1.41–6.44	0.004 *
I lost connection with my relatives or friends in the war	1.88	0.89–3.96	0.09	3.89	1.58–9.53	0.003 *

OR: odds ratio, CI: confidence interval, * *p* < 0.05.

## Data Availability

The data will be available from the corresponding author upon request.
